# Knowledge, Attitude, and Practices Regarding Cosmetics Among Women in the Al-Qunfudah Governorate, Saudi Arabia

**DOI:** 10.7759/cureus.98239

**Published:** 2025-12-01

**Authors:** Safa H Alkalash, Rudhab A Alrizqi, Amnah M Alrashdi, Zeinab M Alanazi, Mariam J Zainab, Reham I Al-sahabi, Salma M Alshreef, Ghadeer Y Almasabi

**Affiliations:** 1 Community Medicine and Health Care, Umm Al-Qura University, Al-Qunfudah, SAU; 2 Family Medicine, Menoufia University, Shebin Elkom, EGY; 3 Medicine and Surgery, Umm Al-Qura University, Al-Qunfudah, SAU; 4 Medicine, College of Medicine, Northern Border University, Arar, SAU; 5 Medicine, Alfaisal University, Riyadh, SAU

**Keywords:** appearance, attitude, cosmetics, knowledge, sunscreen

## Abstract

Background: Cosmetic product use has notably increased among women due to social trends and the growing emphasis on physical appearance. Although cosmetics improve appearance and confidence, they can cause adverse effects ranging from skin irritation to potential systemic harm. Despite these risks, many reactions go unreported.

Objective: This study aimed to assess the knowledge, attitudes, and practices related to cosmetic use and its adverse effects among women in Al-Qunfudah, Saudi Arabia.

Methods: A cross-sectional descriptive study was conducted among 425 women living in Al-Qunfudah. Data were collected over three months, from the first of January 2025 to the end of March 2025, through an online Arabic questionnaire covering demographics, knowledge, attitudes, and cosmetic practices. Knowledge scores were classified as good (≥60%) or poor (<60%). Data analysis was done using IBM SPSS Statistics software, version 27 (IBM Corp., Armonk, NY), with significance set at p<0.05.

Results: Among participants, 289 (68.0%) had adequate knowledge. Significant associations were found between knowledge level and sunscreen use (p=0.005), pharmacist consultation (p=0.012), and cosmetic use (p=0.049). A total of 214 (50.4%) reported side effects, mainly skin-related (184, 86.0%), with hair products as the leading cause (177, 82.7%). The face was the most affected area (165, 80.9%). Most reactions appeared within one month among 166 (77.6%) participants. Despite 78 (46.4%) self-medicating, only 65 (38.7%) sought medical advice.

Conclusion: Although many women in Al-Qunfudah are aware of the importance of product quality and sun protection, there are clear gaps in safe cosmetic practices. These include limited professional consultation, low rates of sensitivity testing, and insufficient awareness of safety regulations. Targeted health education is needed to support safer cosmetic use and reduce the risk of harm.

## Introduction

Cosmetic products have become an essential part of daily life for both men and women due to modernization and the increasing emphasis on appearance. This trend has contributed to the significant annual growth of the global beauty market [1].

Cosmetics are materials designed to be rubbed, poured, sprinkled, sprayed, or injected into any part of the human body in order to clean, beautify, enhance attractiveness, or change the look of that area without changing the structure or function of the body [2]. Based on this definition, cosmetics include products such as soaps, shampoos, skin creams, lotions, perfumes, lipsticks, nail polishes, eye and facial makeup, permanent waves, hair dyes, toothpaste, and deodorants [3]. While cosmetics enhance beauty and boost confidence, they also pose potential risks. Adverse effects may result from individual sensitivity to ingredients, microbial contamination, improper labeling, violations of color additive regulations, or the presence of harmful substances such as preservatives and fragrances [4].

Clinical manifestations of adverse reactions to cosmetics vary and may include itching, corrosive scalp injuries, acute hair loss, cosmetic acne, conjunctivitis, photo-allergic or toxic contact dermatitis, hypo- or hyperpigmentation, and oral mucosal irritation [5-7]. In addition to dermatological concerns, long-term exposure to certain cosmetic ingredients has been linked to potential systemic health effects. Research suggests that some chemical components, such as parabens and phthalates, may act as endocrine disruptors, interfering with hormonal balance and posing potential reproductive health risks [8]. Furthermore, prolonged use of cosmetics containing heavy metals, such as lead in lipsticks and mercury in skin-lightening creams, has been associated with neurotoxicity and other serious health effects [9].

 Despite these risks, documented cases of adverse reactions to cosmetics remain limited. This may be due to the tendency of consumers to self-medicate for mild to moderate reactions rather than seek medical attention [10-11]. A 2006 survey examined 3,474 cosmetic users, of whom 848 reported experiencing adverse reactions to the products. The findings revealed that more than half of the women who experienced negative reactions did not seek medical advice or consult a pharmacist [12].

According to Poranki and Perwej, the use of cosmetics in Saudi Arabia is increasing as income levels rise, which is a noteworthy development and an important factor for marketers looking to enter this market with modern marketing strategies [13]. Recent reports from Saudi Arabia indicate an inadequate level of awareness regarding the adverse effects of cosmetics [14]. Although earlier Saudi research evaluated the general public's understanding of cosmetics, no such studies have been conducted in the Al-Qunfudah governorate, a distant region with little access to medical care. Therefore, the aim of this study was to assess knowledge, attitudes, and practices related to cosmetics and their adverse effects among women in Al-Qunfudah, Saudi Arabia.

## Materials and methods

Study design

A cross-sectional descriptive study was conducted in Al-Qunfudah governorate, Saudi Arabia, and data were collected over three months, from the first of January 2025 to the end of March 2025.

Inclusion Criteria

The study participants had to possess all the following criteria to be recruited in this study: All women 18 years old and older, living in Al-Qunfudah governorate, who gave informed consent to participate in the study.

Exclusion Criteria

Men, and women less than 18 years old living outside Al-Qunfudah governorate were excluded from the study.

The sample size of 425 was determined through the Raosoft calculator (Raosoft Inc., Seattle, WA) [14], based on the Al-Qunfudah governorate's population size of 300,516, with a 5% marginal error and a 95% confidence interval, and the expected response rate is 50% [15].

Data collection

An online Arabic questionnaire was designed using Google Forms (Google Inc., Mountain View, CA), where participants received electronic links through different electronic platforms, such as Telegram (Telegram FZ‑LLC, Dubai, United Arab Emirates) and X (X Corp., Bastrop, TX) applications, with a brief explanation of the study objectives, the target population, and a request to participate voluntarily.

The questionnaire (Appendix A) was created by the study researchers after conducting a literature review [1, 4, 10, 16]. It included items to collect data about participants’ demographic characteristics (six items) and knowledge (11 items), attitudes (seven items), and seven items to evaluate their practice, as well as nine items about adverse reactions after cosmetic use. This questionnaire's validity was pre-tested in a pilot study that included 40 responses, which were collected to test the tool. The data from the pilot study were excluded from the main study results. A Cronbach factor was 0.80 for the overall questionnaire and 0.81, 0.78, 0.79, and 0.80 for knowledge, attitudes, practice, and side effect items, respectively.

Statistical analysis

The data analysis for this study was conducted using IBM SPSS Statistics software, version 27 (IBM Corp., Armonk, NY). Descriptive statistics, including frequencies and percentages, were used for presenting the qualitative data, while quantitative data were presented as the mean and standard deviation (mean ± SD). For the analysis of knowledge and attitudes, the knowledge score was calculated by assigning one point for each correct answer. The knowledge level was categorized into two groups: poor and adequate knowledge, with a cutoff of 60%. Participants who scored below 60% were classified as having poor knowledge, while those scoring 60% or above were categorized as having adequate knowledge. To assess associations between various demographic factors and knowledge, the chi-square (χ²) test was used for categorical variables. In cases where the expected frequency count was low, the exact probability test was employed. Additionally, to compare the differences between groups, the p-value was calculated, and a p-value less than 0.05 was considered statistically significant.

Ethics

Helsinki's 1964 Declaration of the World Medical Association, most recently updated in 2008, was followed in this study. Permission was obtained from Umm Al-Qura University's (UQU) Institutional Review Board; ethical permission was acquired in September 2024 under the number HAPO-02-K-012-2024-10-2269. All participants were asked to provide informed consent for the study before they could answer the questionnaire, guaranteeing that all information would be shared anonymously.

## Results

Out of the 425 respondents, age distribution reveals that 193 participants (45.4%) were in the 18-24 age range, with a mean age of 29.7 years. Just 10 (2.4%) of women had only a basic education, whereas 292 (68.7%) had a university degree or above. Regarding employment, 143 (33.6%) of the women had a job. A total of 307 (72.2%) claimed to have a monthly salary of less than 5000 Saudi riyal (SR), and 29 (6.8%) reported earning more than 15000 (Table 1).

**Table 1:** Sociodemographic characteristics of the study subjects (N=425) SR: Saudi Riyal

Data	N.	%
Age in years (Mean ± SD)	29.7 ± 10.6	
18-24	193	45.4
25-30	90	21.2
31-40	51	12.0
> 40	91	21.4
Educational level		
Basic education	10	2.4
Secondary education	85	20.0
Diploma	38	8.9
University/above	292	68.7
Having a profession		
Yes	143	33.6
No	282	66.4
Monthly income		
< 5000 SR	307	72.2
5000-10000 SR	59	13.9
>10000-15000 SR	30	7.1
> 15000 SR	29	6.8

There was a split in the responses about the first stage in skin cleaning: 133 (31.3%) selected oil, 131 (30.8%) opted for cleansing milk, and 161 (37.9%) were unsure. Most respondents (307, 72.2%) said that perfumes are the main source of skin allergies, followed by jewelry (57, 13.4%) and uncertainty (61, 14.4%). Waterproof mascara was identified by 345 (81.2%) of responders as the most detrimental to eyelashes. A resounding 361 (84.9%) of respondents accurately said that sunscreen prevents skin cancer. Nearly half of the respondents said that the most common negative reaction to chemical dyes is skin dandruff. A total of 358 (84.2%) of respondents accurately identified the usage of oxidants as the main cause of hair dyes' negative effects. For shampoo use after hair dyeing, 357 (84.0%) recommended promoters. Among the participants, 340 (80.0%) correctly identified skin irritation and sensitivity, while 53 (12.5%) chose dryness as the most common side effect of cosmetics and pharmaceuticals on the skin. Regarding the appropriate skin for using cosmetic powders, 302 (71.1%) correctly chose wet skin. 260 (61.2%) mentioned oily cosmetics as being the cosmetic group with the most side effects on the skin; 284 (66.8%) correctly identified discoloration of nails as the common side effect of nail polish. The vast majority (89, 68.0%) had a good level of knowledge about cosmetics, while 136 (32.0%) of the participants had poor knowledge (Table 2).

**Table 2:** Participants’ knowledge regarding cosmetics (N=425)

Item	N.	%
What is the ﬁrst step for cleaning the skin?		
Cleansing milk	131	30.8
Oil	133	31.3
Do not know	161	37.9
What is the most prevalent cause of skin allergy?		
Perfumes	307	72.2
Jewelry	57	13.4
Do not know	61	14.4
What is the most harmful type of mascara for eyelashes?		
Waterproof mascaras	345	81.2
Simple	19	4.5
Do not know	61	14.4
What is the advantage of using sunscreens?		
Skin cancer prevention	361	84.9
Prevention of dust	31	7.3
Do not know	33	7.8
What is the most prevalent side eﬀect of chemical dyes?		
Skin dandruff	217	51.1
Oiliness	60	14.1
Do not know	148	34.8
What is the most important reason for the harmful eﬀects of hair dyes?		
Using oxidants	358	84.2
Using color	14	3.3
Do not know	53	12.5
What is the appropriate shampoo after hair dyeing?		
Promoters	357	84.0
Any shampoo	24	5.6
Do not know	44	10.4
What is the most common side effect of cosmetics and pharmaceuticals on skin?		
Skin irritation and sensitivity	340	80.0
Dryness	53	12.5
Do not know	32	7.5
What is appropriate skin for using cosmetic powders?		
Dry skin	50	11.8
Wet skin	302	71.1
Do not know	73	17.2
What is the cosmetics group that had the most side effects on skin?		
Oily cosmetics	260	61.2
Powders	67	15.8
Do not know	98	23.1
What is the common side effect of nail polish?		
Discoloration of nails	284	66.8
Itching	20	4.7
Do not know	121	28.5
Overall knowledge score		
Good	289	68.0
Poor	136	32.0

Regarding the attitudes towards cosmetics among women in the Al-Qunfudah governorate, a majority of participants (355, 83.5%) believed that improper use of cosmetics has harmful effects on the skin. There was a strong consensus that quality outweighs cost, as 253 (59.5%) strongly agreed and 135 (31.8%) agreed about this issue; 124 (29.2%) strongly agreed, and 127 (29.9%) agreed that tattoos cause cancer. Only 39 (9.2%) agreed, and 17 (4.0%) strongly agreed that laser therapy is carcinogenic. In contrast, 121 (28.5%) disagreed, and 25 (5.9%) strongly disagreed; 159 (37.4%) agreed, while 128 (30.1%) strongly agreed that hair coloring is dangerous to pregnant and breastfeeding mothers; 197 women (46.4%) and 142 (33.4%) strongly agreed that sunscreen use should begin at a young age, suggesting a strong understanding of the necessity of sun protection. Finally, 148 (34.8%) disagreed, with 50 (11.8%) strongly opposing the use of water to clean cosmetics (Table 3).

**Table 3:** Participants' perceptions of cosmetics (N=425)

Attitude	Strongly disagree		Disagree		Neutral		Agree		Strongly agree	
	N.	%	N.	%	N.	%	N.	%	N.	%
Inappropriate use of cosmetics causes rashes, skin darkening, and wrinkles	1	.2	8	1.9	61	14.4	182	42.8	173	40.7
The quality of cosmetics is more important than their price	0	0.0	10	2.4	27	6.4	135	31.8	253	59.5
Tattoo causes cancer	0	0.0	19	4.5	155	36.5	127	29.9	124	29.2
Laser therapy is carcinogenic	25	5.9	121	28.5	223	52.5	39	9.2	17	4.0
Hair dyeing has harmful effects on pregnant and breastfeeding women	4	0.9	30	7.1	104	24.5	159	37.4	128	30.1
Using sunscreens should start from an earlier age	7	1.6	27	6.4	52	12.2	142	33.4	197	46.4
I usually prefer to use water for cleaning cosmetics	50	11.8	148	34.8	55	12.9	121	28.5	51	12.0

A total of 133 women (31.3%) reported using cosmetics, while 281 (66.1%) reported using sunscreens. Only 142 (33.4%) consult pharmacists for selecting cosmetics, while 283 (66.6%) do not. Regarding hair dyeing, 282 (66.4%) considered the continuity of color effects on hair, and 311 (73.2%) focused on the manufacturer's company. Additionally, 302 (71.1%) prioritized the harmlessness of the product, while 335 (78.8%) considered the expiration date. However, only 252 (59.3%) did a sensitivity test before hair dyeing. When choosing face cosmetics, the majority of women were concerned about factors such as manufacturing and consumption data (338, 79.5%) and brand credibility (348, 81.9%). In contrast, 218 (51.3%) paid attention to the production license, while 207 (48.7%) didn't. Around 297 (69.9%) applied nail polish, whereas 339 (79.8%) used acetone to clean it. However, 128 (30.1%) did not use nail polish, and 86 (20.2%) did not use acetone (Table 4).

**Table 4:** Participants' practice regarding cosmetics and related products (N=425)

Practice	Yes		No	
	N.	%	N.	%
Do you use cosmetics?	133	31.3	292	68.7
Do you use sunscreens?	281	66.1	144	33.9
Do you consult pharmacists for selecting cosmetics?	142	33.4	283	66.6
Do you pay attention to the following factors in the selection of dyes for your hair?				
Continuity of color effects on hair	282	66.4	143	33.6
Manufacturing company	311	73.2	114	26.8
Harmlessness	302	71.1	123	28.9
Expiration date	335	78.8	90	21.2
Doing a sensitivity test before hair dying	252	59.3	173	40.7
Do you pay attention to the following factors when selecting face cosmetics?				
Production and consumption data	338	79.5	87	20.5
Production license	218	51.3	207	48.7
Brand credibility	348	81.9	77	18.1
Do you use nail polishes?	297	69.9	128	30.1
Do you use acetone for cleaning nail polishes?	339	79.8	86	20.2

A total of 214 study subjects (50.4%) experienced cosmetic side effects. Of them, 177 (82.7%) attributed side effects to haircare products, while 37 (17.3%) identified skincare products as the source of their reactions; 166 (77.6%) reported that their reactions occurred within one month, while 48 (22.4%) experienced side effects within a year of cosmetics usage. The most prevalent form of reaction was a skin reaction alone, which affected 184 women (86.0%), followed by systemic reactions in 21 (9.8%), and a combination of skin and systemic reactions in nine (4.2%). The affected body areas were predominantly the face, by 165 (80.9%), followed by the hair and eyes (12.3% each), and the hands (11.8%). For those who sought consultation for the side effects, 65 (38.7%) consulted a dermatologist, while 16 (9.5%) consulted a pharmacist, and 78 (46.4%) chose to self-medicate. A smaller number, 9 (5.4%), sought advice from a beautician. 109 (55.1%) stopped using the product until the adverse events disappeared, 41 women (20.7%) changed the product, and 36 (18.2%) used drug treatment. A smaller group, 12 (6.1%), withdrew the product entirely. In terms of recovery, 185 (86.4%) reported that the side effects completely subsided after treatment, while 29 (13.6%) did not experience full recovery (Table 5).

**Table 5:** Adverse consequences of cosmetics use among research participants (N=425)

Side effects	N.	%
Did you previously experience side effects of cosmetics use? (n= 425)		
Yes	214	50.4
No	211	49.6
What was the type of cosmetic product used? (n= 214)		
Skincare	37	17.3
Haircare	177	82.7
What was the duration of the reaction that occurred? (n= 214)		
Within 1 month	166	77.6
Within 1 year	48	22.4
What was the type of reaction? (n= 214)		
Skin reaction only	184	86.0
Systemic reaction only	21	9.8
Skin and systemic reactions	9	4.2
What was the affected body area? (n= 214)		
Face	165	80.9
Hair	25	12.3
Eyes	25	12.3
Hand	24	11.8
Scalp	17	8.3
Lips	15	7.4
Nose	14	6.9
Legs	9	4.4
Upper limbs	3	1.5
Lower limbs	1	0.5
What was the type of consultation adopted? (n= 214)		
Dermatologist	65	38.7
Pharmacist	16	9.5
Started self-medication	78	46.4
Beautician	9	5.4
What was the nature of management adopted?		
Product withdrawal	12	6.1
Product change	41	20.7
Drug treatment	36	18.2
Stopped using the product until adverse events disappeared	109	55.1
Did the side effects completely subside after treatment?		
Yes	185	86.4
No	29	13.6
How long did it take for the side effect to subside?		
Within 1-2 weeks	116	68.6
Within 3-4 weeks	30	17.8
More than 1 month	23	13.6

A higher proportion of non-cosmetic users, 207 (70.9%), had good knowledge, suggesting that those who used cosmetics had slightly lower knowledge levels, with a p-value of 0.049. Sunscreen use was also significantly associated with better knowledge. Among sunscreen users, 204 (72.6%) had good knowledge, while only 59 (59.0%) of non-sunscreen users reported good knowledge. Additionally, 59 (41.0%) of non-users had poor knowledge, compared to 77 (27.4%) of sunscreen users, and the p-value was 0.005. Another significant factor was consulting pharmacists for selecting cosmetics. Among those who consulted pharmacists, 108 (76.1%) had good knowledge, compared to 181 (64.0%) among those who did not. The p-value is 0.012. Other factors, such as age, educational level, having a profession, and monthly income, did not show significant associations with overall knowledge, as their p-values were greater than 0.05 (Table 6).

**Table 6:** Factors associated with the participants' knowledge about cosmetics (N=425) P: Pearson X^2^ test; ^: Exact probability test; P < 0.05 (significant); SR: Saudi Riyal

Factors	Overall knowledge level				Test	p-value
	Poor		Good			
	N.	%	N.	%		
Age, in years					6.138	0.105
18-24	60	31.1	133	68.9		
25-30	26	28.9	64	71.1		
31-40	12	23.5	39	76.5		
> 40	38	41.8	53	58.2		
Educational level					5.113	0.164^
Basic education	5	50.0	5	50.0		
Secondary education	34	40.0	51	60.0		
Diploma	12	31.6	26	68.4		
University / above	85	29.1	207	70.9		
Having a profession					0.075	0.785
Yes	47	32.9	96	67.1		
No	89	31.6	193	68.4		
Monthly income					2.160	0.540^
< 5000 SR	103	33.6	204	66.4		
5000-10000 SR	18	30.5	41	69.5		
>10000-15000 SR	9	30.0	21	70.0		
> 15000 SR	6	20.7	23	79.3		
Do you use cosmetics?					3.583	0.049
Yes	51	38.3	82	61.7		
No	85	29.1	207	70.9		
Do you use sunscreens?					8.057	0.005
Yes	77	27.4	204	72.6		
No	59	41.0	85	59.0		
Do you consult pharmacists for selecting cosmetics?					6.361	0.012
Yes	34	23.9	108	76.1		
No	102	36.0	181	64.0		

Age is the only significant factor associated with the use of cosmetics among women in the Al-Qunfudah governorate, with a p-value of 0.042. Among women aged 18-24, 73 (37.8%) used cosmetics, while 120 (62.2%) did not. No significant associations were found between cosmetic use and other factors, such as educational level (p = 0.352), having a profession (p = 0.543), or monthly income (p = 0.509) (Table 7).

**Table 7:** Distribution of cosmetic use according to the demographic characteristics of the study participants P: Pearson X^2^ test; ^: Exact probability test; P < 0.05 (significant); SR: Saudi Riyal

Factors	Do you use cosmetics?				Test	p-value
	Yes		No			
	N.	%	N.	%		
Age, in years					8.185	0.042
18-24	73	37.8	120	62.2		
25-30	27	30.0	63	70.0		
31-40	12	23.5	39	76.5		
> 40	21	23.1	70	76.9		
Educational level					3.267	0.352^
Basic education	3	30.0	7	70.0		
Secondary education	33	38.8	52	61.2		
Diploma	13	34.2	25	65.8		
University/above	84	28.8	208	71.2		
Having a profession					0.371	0.543
Yes	42	29.4	101	70.6		
No	91	32.3	191	67.7		
Monthly income					2.317	0.509^
< 5000 SR	101	32.9	206	67.1		
5000-10000 SR	17	28.8	42	71.2		
>10000-15000 SR	6	20.0	24	80.0		
> 15000 SR	9	31.0	20	69.0		

## Discussion

The results of the current study, which evaluated the level of cosmetics knowledge among Saudi Arabian women in Al-Qunfudah, are quite intriguing. Many women are aware that skin allergies can be brought on by perfumes, a phenomenon that is also observed in other regions of the world. For example, according to a Canadian study [17], perfumes are a major cause of skin allergies worldwide. To concentrate on giving women greater knowledge, doctors are becoming more aware of what they already know about cosmetics. Similarly, the recognition of waterproof mascaras as the most harmful type for eyelashes reflects a growing global awareness of the damaging effects of certain cosmetic products, as noted in a 2020 study in the United States [18]. However, the relatively high percentage of participants who were unsure about the first step in skin cleaning or the side effects of chemical dyes suggests a need for targeted educational campaigns, a finding reported in a 2018 study in the west of Saudi Arabia, which emphasized the lack of public awareness about cosmetic ingredients and their potential risks [19].

The awareness of the role of sunscreens in preventing skin cancer is particularly notable, as it reflects a positive trend in public health knowledge. This matches with global efforts to promote sunscreen use, such as the World Health Organization’s campaigns on UV radiation protection [20]. However, the small but significant percentage of women who believed sunscreens prevent dust or were unsure about their benefits indicates lingering misconceptions. These findings are consistent with a 2024 study in Aseer, Saudi Arabia, which found that while most women understood the importance of sunscreens, a subset held incorrect beliefs about their primary function [15]. Similarly, a study by Nohynek et al. [21] identified oxidants as the main hazardous component in hair dyes. However, the lack of knowledge about the right shampoo to use after dyeing one's hair reveals a lack of practical cosmetic education, which was previously identified in a 2015 study carried out in Dubai [22].

The high level of awareness regarding skin irritation and sensitivity as the most common side effect of cosmetics and pharmaceuticals is promising, as it reflects a growing understanding of skin health. This finding is supported by a 2019 study in Egypt, which reported similar levels of awareness among women in Cairo [23]. However, the misconceptions about the appropriate skin type for cosmetic powders and the side effects of nail polish highlight areas where knowledge is lacking. For example, the belief that dry skin is suitable for cosmetic powders contradicts dermatological recommendations, which emphasize the importance of matching products to skin type [24]. This discrepancy is also noted in a 2011 study in Kuwait, where similar misconceptions were observed [25]. Overall, while most women in Al-Qunfudah demonstrated good knowledge of cosmetics, the persistent gaps in understanding suggest a need for more comprehensive and accessible educational resources, particularly in regions with limited access to dermatological expertise.

The results from Al-Qunfudah governorate reveal a pattern of knowledge that is both consistent with and divergent from findings in other regions. The high levels of awareness in certain areas, such as sunscreen use and the harmful effects of waterproof mascaras, reflect global trends in cosmetic education. However, the gaps in knowledge, particularly regarding skin cleaning steps and the side effects of chemical dyes, highlight the need for targeted interventions. These findings underscore the importance of culturally tailored public health campaigns to address misconceptions and improve cosmetic literacy, not only in Saudi Arabia but also in other regions with similar demographic and educational profiles.

In line with reported worldwide concerns, a significant proportion of women are aware that inappropriate cosmetic use can have adverse consequences such as rashes, skin darkening, and wrinkles [26, 27]. In addition, there is a definite preference for quality over pricing, which is indicative of a larger movement to put product efficacy and safety first. Uncertainty surrounds the health risks associated with tattoos, and there are different opinions about the carcinogenicity of laser therapy, indicating a need for improved public education. Conversely, there's a strong consensus regarding the harmful effects of hair dye during pregnancy and breastfeeding, and a high level of awareness regarding the importance of sunscreen use. Nevertheless, varying preferences for water as a cosmetic cleaning method suggest a need for clearer guidelines on effective cosmetic removal practices. While women demonstrate a general understanding of cosmetic risks and prioritize safety, misconceptions persist regarding specific treatments and basic cosmetic practices, highlighting the importance of targeted educational initiatives.

Considering practice, both positive behaviors and areas requiring improvement were also reported. A significant proportion of women use sunscreens. However, the limited consultation with pharmacists for cosmetic selection suggests a tendency to rely on personal preferences rather than professional advice. When choosing hair dyes, women prioritize factors like color continuity and product safety, consistent with observations in many studies [28-29]. Yet, a concerning number do not perform sensitivity tests before utilizing hair dye. Similarly, while brand credibility and production data are considered for face cosmetics, the focus on production licenses is relatively low, indicating a need for greater awareness of regulatory standards. Furthermore, the prevalent use of nail polish and acetone for removal, while common, raises concerns about potential health risks, such as nail brittleness and skin irritation, augmenting the need for safer alternatives to acetone, as noted in a U.S. study [30].

In brief, the findings reveal that cosmetic use, sunscreen use, and consulting pharmacists significantly influence women's cosmetic knowledge in Al-Qunfudah. Notably, cosmetic users demonstrated slightly lower knowledge levels compared to non-users, while sunscreen users and those who consulted pharmacists exhibited significantly better knowledge. Age was the only demographic factor significantly associated with cosmetic use, with younger women more likely to use cosmetics than older women. Other demographic factors like education, profession, and income did not show significant associations with either knowledge or cosmetic use. Essentially, practical behaviors like sunscreen use and seeking professional advice correlated with better knowledge, while age influenced cosmetic usage patterns.

Strengths and limitations

This study has several strengths, including being conducted in an underexplored population (Saudi women in Al-Qunfudah) and employing a validated questionnaire that is simple and clear and enhances accessibility. The second strength of this study is that it was conducted on a large sample size, and its topic has strong community relevance. However, several restrictions need to be mentioned. The cross-sectional study limits causal inferences, and the dependence on self-reported data raises concerns about bias. The study included women 18 and older, but cosmetics may also be used by adolescent females. It was more appropriate to include men, as their knowledge is important because some of them can use materials like sunscreen, and their awareness could influence female relatives. Furthermore, the study's geographical focus may limit generalizability, highlighting the importance of including a more diversified sample.

## Conclusions

In conclusion, this study revealed a mix of moderate women's knowledge and variable attitudes and practices regarding cosmetics in Al-Qunfudah, Saudi Arabia. While there's strong awareness of sunscreen and quality product selection, gaps exist in professional consultation, sensitivity testing, and understanding regulatory standards. Also, women in Al-Qunfudah prioritize safe and high-quality cosmetics; gaps in consulting professionals, performing sensitivity tests, and understanding regulatory standards emphasize the necessity of focused educational initiatives to encourage safer cosmetic procedures. To improve, targeted education campaigns are crucial, emphasizing professional guidance, safety practices, and safer product alternatives. By addressing these gaps, women can make informed choices for safer cosmetic use.

## Appendices

Appendix A 

**Table 8:** Questionnaire for assessment of knowledge, attitude, and practice regarding cosmetics among women in Al-Qunfudah Governorate, Saudi Arabia Credits: the authors; Safa H. Alkalash, Rudhab A. Alrizqi, Amnah M. Alrashdi, Zeinab M. Alanazi, Mariam J. Zainab, Reham I. Al-sahabi, Salma M. Alshreef, Ghadeer Y. Almasabi

Do you agree to participate in this study?											
Yes											
No											
Demographic:											
What is your area of residency?											
Al-Qunfudah governorate											
Outside Al-Qunfudah governorate											
What is your gender?											
Male											
Female											
What is your age in years?											
What is your education level?											
Intermediate school or below											
Secondary school											
Diploma											
University and above											
Do you have an occupation?											
Yes											
No											
What is your monthly income (in Saudi Riyal)?											
Less than 5000											
5000-10000											
>10000-15000											
>15000											
Knowledge-based questions											
What is the ﬁrst step for cleaning the skin?											
Oil											
Cleansing milk											
Do not know											
What is the most prevalent cause of skin allergy?											
Perfumes											
Jewelry											
Do not know											
What is the most harmful type of mascara for eyelashes?											
Simple											
Waterproof mascaras											
Do not know											
What is the advantage of using sunscreens?											
Prevention of dust											
Skin cancer prevention											
Do not know											
What is the most prevalent side eﬀect of chemical dyes?											
Oiliness											
Skin dandruff											
Do not know											
What is the most important reason for the harmful eﬀects of hair dyes?											
Using color											
Using oxidants											
Do not know											
What is the appropriate shampoo after hair dyeing?											
Promoters											
Any shampoo											
Do not know											
What is the most common side effect of cosmetics and pharmaceuticals on skin?											
Dryness											
Skin irritation and sensitivity											
Do not know											
What is appropriate skin for using cosmetic powders?											
Wet skin											
Dry skin											
Do not know											
What is the cosmetics group that had the most side effects on skin?											
Powders											
Oily cosmetics											
Do not know											
What is the common side effect of nail polish?											
Itching											
Discoloration of nails											
Do not know											
Attitude-based questions											
Inappropriate use of cosmetics causes rashes, skin darkening, and wrinkles											
Strongly Agree	Agree	Neutral		Disagree	Strongly Disagree						
The quality of cosmetics is more important than their price											
Strongly Agree	Agree	Neutral		Disagree	Strongly Disagree						
Tattoo causes cancer											
Strongly Agree	Agree	Neutral		Disagree	Strongly Disagree						
Laser therapy is carcinogenic											
Strongly Agree	Agree	Neutral		Disagree	Strongly Disagree						
Hair dyeing has harmful effects on pregnant and breastfeeding women											
Strongly Agree	Agree	Neutral		Disagree	Strongly Disagree						
Using sunscreen should start from an early age											
Strongly Agree	Agree	Neutral		Disagree	Strongly Disagree						
I usually prefer to use water for cleaning cosmetics											
Strongly Agree	Agree	Neutral		Disagree	Strongly Disagree						
Practice-based questions											
Do you use cosmetics?							Yes			No	
Do you use sunscreens?							Yes			No	
Do you consult pharmacists to select cosmetics?							Yes			No	
Do you pay attention to the following factors in the selection of dye hair?											
Continuity of color effects on hair						Yes					No
The manufacturer company						Yes					No
Harmlessness						Yes					No
Expiration date						Yes					No
Doing a sensitivity test before hair dying						Yes					No
Do you pay attention to the following factors in selecting face cosmetics?											
Production and consumption data								Yes	No		
Production license								Yes	No		
Brand credibility								Yes	No		
Do you use nail polishes?								Yes	No		
Do you use acetone for cleaning nail polishes?								Yes	No		
Did you experience side effects from cosmetic use?								Yes	No		
If yes, please answer the following questions											
What was the type of cosmetic product?											
Skincare											
Haircare											
Not applicable											
What was the duration of the reaction that occurred?											
Within 1 week											
Within 1 month											
Within 1 year											
Not applicable											
What was the type of reaction?											
Skin reaction only (local itching or redness)											
Systemic reaction only (itching, redness, shortness of breath, or systemic symptoms)											
Skin and systemic											
Not applicable											
What was the affected body area?											
Face			Eyes								
Hair			Hand								
Legs			Lips								
Scalp			Lower limbs								
Upper limbs			Nose								
Not applicable			None								
What was the type of consultation adopted?											
Dermatologist											
Pharmacist											
Started self-medication											
Beautician											
Not applicable											
What was the nature of management adopted?											
Product withdrawal											
Product change											
Drug treatment											
Stopped using the product until adverse events disappeared											
Not applicable											
Did the side effects completely subside after treatment?											
Yes											
No											
How long did it take for the side effect to subside?											
Within 1-2 weeks											
Within 3-4 weeks											
More than 1 month											
Not applicable											
